# Narrative and visual attention in autism spectrum disorder: a cross-cultural perspective

**DOI:** 10.3389/fpsyt.2026.1589600

**Published:** 2026-06-10

**Authors:** Jiayin Xing, Kritika Nayar, Emily Landau, Xin Kang, Joseph C. Y. Lau, Cassandra Stevens, Gary E. Martin, Patrick C. M. Wong, Molly Losh

**Affiliations:** 1 Roxelyn and Richard Pepper Department of Communication Sciences and Disorders, Northwestern University, Evanston, IL, United States; 2 Department of Child and Adolescent Psychiatry, Child Study Center, Hassenfeld Children’s Hospital at NYU Langone, New York, NY, United States; 3 Department of Linguistics and Modern Languages, The Chinese University of Hong Kong, Hong Kong, Hong Kong SAR, China; 4 Brain and Mind Institute, The Chinese University of Hong Kong, Hong Kong, Hong Kong SAR, China; 5 Research Centre for Language, Cognition, and Language Application, Chongqing University, Chongqing, China; 6 Kansas Center for Autism Research and Training, Life Span Institute, University of Kansas, Lawrence, KS, United States; 7 Department of Clinical Child Psychology, University of Kansas, Lawrence, KS, United States; 8 Department of Communication Sciences and Disorders, St. John’s University, Queens, NY, United States

**Keywords:** ASD, cross-culture, eye-tracking, gaze, narrative

## Abstract

**Introduction:**

Narrative, or storytelling, is a primary form of social communication. Prior work has documented differences in narrative in individuals with autism spectrum disorder (ASD), but predominantly focused on English-speaking Western countries. Given the internalization of cultural values and conventions shapes narrative, the extent to which environmental (i.e., cultural and linguistic) factors may interact with ASD to influence the narrative ability remains unclear. The current study applied a cross-cultural approach to investigate narrative quality in ASD between a Western (United States) culture and an Eastern [Chinese (Hong Kong)] culture. Given that visual attention and exploration inform one’s experiences and consequently storytelling, visual attention during narration was examined to unravel underlying mechanisms contributing to narrative.

**Methods:**

Participants included 56 autistic individuals (US-ASD) and 49 non-autistic controls (US-control) from the US who were native English speakers, as well as 24 autistic individuals (HK-ASD) and 52 controls (HK-control) from the Hong Kong SAR, China (HK), who were native Cantonese speakers, with age matched across groups. Participants narrated a wordless picture book presented on an eye-tracker, with narrative quality and dynamic gaze patterns examined.

**Results:**

Group comparisons revealed that autistic individuals from both cultures missed key story components and exhibited elevated rigid gaze patterns towards social stimuli. Cultural variations were also demonstrated, with US-ASD group, but not HK-ASD group, narrating with fewer descriptions and causal attributions of characters’ thoughts/emotions. Further, correlational analyses revealed the association between decreased social attention and fewer descriptions of characters’ thoughts/emotions across diagnostic and cultural groups.

**Discussion:**

Together, the findings highlight a difficulty in overall narrative structure and a pattern of regressive/rigid social attention present in ASD across cultures. In contrast, the difficulty in usage of cognitive/emotional terms and causal attributions in ASD appear to be culturally influenced, and were only present in US-ASD. Gaze-narrative associations detected across diagnosis and cultures emphasize the importance of extrapolating underlying mechanistic processes contributing to social language use in general and in ASD. Findings implicate key features of narrative ability that may be more or less malleable to cultural influences, with implications for the biological underpinnings of ASD and culturally-centered clinical practices.

## 1 Introduction

Narrative, or storytelling, is a primary form of social communication used to organize events in temporal and causal frameworks to communicate one’s reality during social interactions (1, 2). Hence, narrative skill is also of great importance to the development of higher-level language abilities (e.g., reading comprehension; 3), mental schema (e.g., self-representation; 4, 5), and social relationships (e.g., 6, 7).

Important aspects of narrative competence are impacted in individuals with autism spectrum disorder (ASD), a condition characterized by social communicative deficits and restricted and repetitive behaviors (8)^1^. Difficulty in narrative structure (i.e., overall content and hierarchical organization) and narrative evaluation (i.e., narrator’s subjective interpretations of events, relationships, and character intent and emotions) have been consistently observed in ASD (6, 9–15). Specifically, autistic individuals have been found to include fewer key story components than controls (6, 16, 17), and show difficulties in establishing the story’s theme (e.g., 18). This difference in narrative structure has also been observed across linguistic backgrounds other than English (e.g., Finnish, Mandarin, German, Hebrew, Greek, French; 19–25). ASD-specific difficulties in narrative evaluation have also been reported. Specifically, autistic individuals produced narratives with fewer evaluative devices (both frequency and type) relative to controls, such as fewer descriptions of thoughts and emotions of the story characters (6, 26, 27) and causal attributions (6, 10, 28). These narrative differences in ASD impact narrative coherence, which can undermine broader social communication skills.

To understand the potential roots of narrative differences in ASD, a body of studies have focused on understanding visual attention patterns during narration. Gaze patterns during narration, including both broad scanning of a story’s scene and focused examination of individual characters or features, may reflect the cognitive process of the narrator (e.g., 29, 30). Specifically, visual scanning patterns may provide insights into how one interprets a story’s details to understand and convey the gist of a narrative, and to interpret the actions and mental states of individual story characters (e.g., 29–32). Recent work has suggested that autistic individuals may demonstrate differences in visual attention during language tasks including narrative (33, 34). Nayar and colleagues examined gaze during a rapid automatized naming task and found ‘sticky’ eye-movement patterns in ASD (i.e., increased repeated fixations on the same stimuli), which was associated with decreased narrative quality measured in another structured picture-book task (34). The study suggested that rigid and repetitive fixation patterns may be one potential mechanism contributing to narrative difficulties in ASD, such that reduced exploration of the whole scene may limit an understanding of the relationships between story characters and events, as well as the overall theme of the story. Besides the visual scanning patterns, the distribution of attention or visual focus may also relate to narrative quality. Lee and colleagues found that when viewing story images, autistic individuals exhibited increased attention to the most prominent and detailed features of the images, while decreasing their focus on more inconspicuous background elements, which correlated with their increased narrative quality (33). These studies highlighted how dynamic visual attention may play an important role in shaping narrative content and coherence for autistic individuals. The current study sought to build on this prior work by examining more dynamic gaze patterns during storytelling and investigating how these visual attention processes relate to more detailed aspects of narrative structure and evaluation in autism.

Despite the widely documented differences in narrative in ASD, prior studies have mostly examined English-speaking individuals from Western countries, creating a critical gap in the literature on the impact of cultural and linguistic backgrounds on narrative competence in ASD. Considerable evidence suggests that cultural environments have important influences on narrative based on the socio-constructivist view of learning, such that conventions from social activities and interactions from one’s cultures are internalized to form their own narratives (35–37).

Narrative evaluation has been found to be particularly influenced by cultural factors (e.g., 38, 39). For example, prior work in non-autistic individuals found that compared to individuals from the US, those from Asia (38–41) tended to avoid making explicit evaluative comments (e.g., mothers were less likely to talk about emotions of story characters in shared reading). From an educational perspective, compared with education in the US, education in Asian countries have a relatively lower emphasis in creative or imaginative thinking, which is essential for generating evaluation (38, 42). Additionally, while the European-American culture values self-expression (43, 44), Asian cultures tend to view emotion expression as less appropriate (44), with greater emphasis on emotional self-restraint and attentiveness to others (43, 45). In contrast to cultural differences in narrative evaluation, narrative structure has been identified as relatively resistant to different cultural and linguistic environments. Prior studies have found that the inclusion of important story components and establishment and maintenance of the story theme (e.g., 46, 47, resectively) were similar across cultures. Together, these studies indicated that although narrative evaluation, especially emotional expression, may be influenced by cultural values, non-autistic people across different cultural backgrounds displayed comparable skills in encoding the structure and content of the narrative.

Although no direct cross-cultural comparisons of narrative abilities in ASD have been conducted in prior research to our knowledge, findings from studies of non-English-speaking autistic individuals may provide valuable insights into possible cultural differences. For example, no differences between Mandarin-speaking autistic children and controls were found in the use of evaluation terms describing emotions, cognition, desire, or perception in storytelling (19, 48). This lack of group differences is not surprising given the generally lower levels of narrative evaluation documented in individuals from Asian cultural backgrounds (e.g., 38–40). These findings stand in contrast to the narrative differences observed between English-speaking autistic individuals (e.g., 9, 10, 15) and their peers in Western European cultural contexts (e.g., Germany and Finland; 20, 21), who showed differences in narrative evaluation.

Despite the insights provided by studies conducted within single cultural and linguistic environments, direct cross-cultural comparisons are needed using consistent narrative measures and coding systems among groups matched on age and cognitive and language abilities. To address this gap, the present study compares narratives produced by autistic and non-autistic participants from two distinct cultural and linguistic contexts: individuals from the Hong Kong SAR, China (HK) whose native language is Cantonese and individuals from the United States (US) whose native language is English. These contexts were selected because they differ along well-documented sociocultural dimensions, particularly individualistic versus collectivistic orientations (e.g., 49–51) and narrative socialization practices that shape children’s use of internal state language (e.g., 38–40). In addition to sociocultural variation, Cantonese and English differ in fundamental linguistic properties, including morphosyntactic structure, discourse organization, and the grammatical encoding of tense and aspect (52–54). Furthermore, Cantonese has been characterized as more topic-prominent in its discourse organization, in contrast to English’s subject-prominent structure (52). Cross-linguistic research demonstrates that this contrast may shape how events are temporally structured and encoded in narrative discourse (55, 56). At the same time, both contexts are highly urbanized and industrialized societies, permitting relatively controlled comparisons across matched groups. Examining similarities and differences in narrative abilities across these contexts will help clarify aspects of narrative that reflect core features of autism versus those that may be shaped by sociocultural and linguistic influences, which will enable discovery of specific targets for pragmatic intervention that are culturally sensitive.

Overall, the current study aimed to compare key narrative features and associated visual attentional patterns in ASD across the US and Chinese (Hong Kong) (HK) cultures. Specifically, linguistic markers (i.e., narrative structure and evaluation), dynamic gaze patterns during narration, and their associations were examined across diagnostic and cultural groups (i.e., US-ASD, US-control, HK-ASD, and HK-control). We hypothesized that narrative structure differences in ASD would be culturally invariant, such that autistic participants across the US and Chinese (Hong Kong) (HK) cultures would demonstrate reduced narrative structure than their respective non-autistic controls. In contrast, narrative evaluation, particularly the use of internal state language, was expected to be more culturally sensitive. Specifically, we predicted reduced use of internal state language in the US-ASD group relative to US-controls, but no significant diagnostic difference within the HK sample. We also predicted that autistic individuals would exhibit a visual scanning profile reflective of local or detailed processing, which would in turn relate to reduced narrative quality. Because prior work on visual scanning patterns in ASD has been conducted primarily in Western samples (e.g., 57), analyses examining potential cultural context effects in gaze patterns and their associations with narrative outcomes were considered exploratory.

## 2 Materials and methods

### 2.1 Participants

Participants included 56 autistic individuals (US-ASD) and 49 controls (US-control) from the US who spoke English as their first language, as well as age-matched 24 autistic individuals (HK-ASD) and 52 controls (HK-control) from HK who spoke Cantonese as their first language. All participants were verbally fluent in their native languages. This study used a combination of previously and newly reported data. For the US groups, all narrative and gaze variables have been previously reported (33, 34, 58, 59), and are re-presented here to allow direct cross-cultural comparison. For the HK groups, only one gaze variable (i.e., fixation count; see below) had been previously reported (57). Unique to this study is the inclusion of more nuanced analytics of gaze (i.e., refixations and transition of fixations; see below), which significantly expands upon prior work and provides a detailed assessment of visual patterns that may be more culturally sensitive. The narrative data from the HK sample, the Cantonese narrative coding scheme, and all narrative variables for the HK sample are newly reported in this study. The new analyses of associations between narrative abilities and attentional patterns for the HK sample are also novel, following prior analyses conducted with the US sample (59). Where specified, we also applied new analytic approaches to the combined datasets to address the study’s cross-cultural questions.

Participants were recruited from registries and local resources in the Greater Chicago area and Hong Kong. Inclusion criteria required verbal fluency in participants’ native language (English for US participants; Cantonese for HK participants) as well as a diagnosis of ASD for research purposes as follows: for the US groups, diagnostic confirmation was conducted using the Autism Diagnostic Observation Schedule–Second Edition (ADOS-2) (60) and/or the Autism Diagnostic Interview–Revised (ADI-R) (61); for the HK groups, diagnostic status was reported by parents, clinics, or schools and confirmed with the administration of the ADOS-2 (60). ADOS-2 average severity scores indicated that both HK-ASD and US-ASD groups showed a range of symptom severity from low to high, with no significant cultural difference in overall severity levels (HK-ASD: *M* = 5.41, *SD* = 2.65; US-ASD: *M* = 6.65, *SD* = 2.21) (see **Table 1**). Exclusion criteria included: 1) based on parent report, a family history of genetic disorders related to ASD (e.g., fragile X syndrome, dyslexia, or brain injury); 2) low language abilities, including verbal IQ (VIQ) as measured by Wechsler Intelligence Scale for Children—Third Edition (WISC-III) or the Wechsler Abbreviated Scale of Intelligence (WASI) (62, 63) below 80 for participants in the US groups, or impaired language development assessed by Hong Kong Cantonese Oral Language Assessment Scale (HKCOLAS) (i.e., scored ≥1.25 standard deviations below age-normed means on two or more HKCOLAS subtests) (64) in the HK group (see also demographic information presented in **Table 1**).

**Table 1:** Sample characteristics.

Sample characteristics	Cultural and Diagnostic Groups (M (SD)[Range])				Culture effect		Diagnosis effect	
	HK-control (n = 52)	HK-ASD (n = 24)	US-control (n = 49)	US-ASD (n = 56)	*F*/χ^2^	*p*	*F*/χ^2^	*p*
Gender (M/F)	30/22	21/3	25/24	47/9	0.002	0.96	17.75	<.001***
Age (years)	16.63 (7.62)	19.58 (9.76)	19.04 (5.34)	18.34 (6.25)	1.08	0.3	0.75	0.39
	[9-32]	[6-33]	[11-32]	[10-35]				
PIQ	111.45 (11.45)	107.79 (11.74)	113.52 (14.03)	104.18 (15.77)	0.63	0.43	12.28	.001***
	[82-128]	[84-127]	[79-143]	[68-131]				
ADOS-2 Average Severity Score	–	5.41 (2.65)	–	6.65 (2.21)	3.48	0.07	–	–
		[1-10]		[1-10]				

Sample size reflects image with maximum number of participants. PIQ refers to performance or non-verbal IQ. ADOS-2 referes to Autism Diagnostic Observation Schedule, Second Edition. ***p <.001.

Informed assent/consent was obtained from all participants and guardians (as applicable), and procedures were approved by the respective institution’s ethics committee. The Nonverbal Intelligence—Fourth Edition (TONI-IV; 65) was used to measure IQ for participants from HK, and WISC-III or WASI (62, 63) was used to measure IQ for participants from US. Following prior literature (57, 66), TONI-IV nonverbal IQ scores and Performance IQ (PIQ) scores on WISC/WASI were highly comparable and correlated and thus were collapsed for analyses.

### 2.2 Procedures

Narrative samples were elicited using the 24-page wordless picture book, Frog, Where Are You? (67), which was presented on an eye tracker (see Lee et al. (33) and Nayar et al. (57) for details). The story depicts the adventures of a boy and his pet dog searching for their missing frog. The task offers a structured, nonverbal elicitation context that facilitates direct comparison across participants and cultural groups. Because it contains no written text, it minimizes linguistic scaffolding and translation-related variability, making it well suited for cross-linguistic research. The stimulus has been widely used in narrative studies across diverse languages and cultural contexts, including East Asian populations (e.g., 55, 68). Its visual format provides a common event sequence while still allowing participants to construct narratives shaped by their cultural frameworks. Participants were instructed to narrate the story page-by-page in English (for the US groups) or in Cantonese (for the HK groups); the examiner advanced pages after participants finished their narration on each page. Narrative speech was recorded by an external microphone, and gaze patterns during narration were recorded on the eye-tracker, with the whole procedure videotaped. All experiments across sites were performed according to the same guidelines and regulations to ensure comparable data collection procedures.

### 2.3 Narrative transcription

Cantonese narrative samples were transcribed using the Computerized Language Analysis (CLAN) software (69). For the English narrative samples, Systematic Analysis of Language Transcripts (SALT) (70) conventions were used for transcription in either SALT or the EUDICO Linguistic Annotator (ELAN) software platforms (71). All transcribers were trained to establish ≥ 80% word reliability and were blind to group status. Overall, 15% of transcripts were randomly selected for double-transcription for reliability purposes, with roughly equal representations of diagnosis, culture, and sex. Word-level inter-rater reliabilities were 95.9% for Cantonese transcripts and 94.94% for English transcripts.

### 2.4 Story length analysis

Word count was extracted as a measure of story length and used as a denominator to create proportion scores (i.e., *Affect* and *Cognition*, and *Causal Inferences*) (see below for details) or used as a covariate where appropriate (i.e., *Story Components Present*). Before the extraction of word count, examiner utterances, word repetitions, reformulations, and transcription codes were removed from both English and Cantonese transcripts. For English transcripts, the Linguistic Inquiry and Word Count (LIWC; 72) was used to extract word count. For Cantonese transcripts, word count was extracted after automatic word segmentation in SPPAS software, a scientific computer software tool for automatic linguistic annotation and analysis (73, 74). Word segmentation is challenging in Cantonese due to the lack of clear word boundaries (74). To test the reliability of the SPPAS software in our samples across diagnostic groups, we replicated procedures in Fung and Bigi (74) in 20% of randomly selected Cantonese transcripts, with equal representation of sex and diagnostic groups. The word segmentation results from SPPAS software were compared to manual word segmentation by native Cantonese speakers based on published Cantonese corpus (75) and definitions of words in Cantonese linguistics (54). The reliability was 93.75%, which was comparable to that reported in Fung and Bigi (74) and those of other automatic word segmentation algorithms (e.g., 76).

### 2.5 Narrative hand coding

To assess narrative quality, we utilized a English narrative coding scheme developed based on prior studies (6, 55, 59, 77–79), which examines narrative structure and evaluation, as well as some secondary abilities. The assessments of narrative structure included the inclusion of important story elements, and the establishment and maintenance of story theme. The narrative evaluation skills were assessed as the narrators’ abilities to interpret story characters’ thoughts/emotions. The coding scheme was then adapted for Cantonese analysis.

#### 2.5.1 Story structure

The frog story is composed of story elements including the setting, plot instantiation, 5 search episodes (2 search sub-episodes each), and resolution. See **Table 2** for detailed descriptions of each story component.

**Table 2:** Story components.

Search episodes (SE)	Search sub-episodes	Description	Pages
Setting	–	Boy, dog, frog in the bedroom at night	1
Plot Instantiation	–	Frog escaping from the jar	2-3
SE1	SE1.1	Boy and dog searching for frog in the bedroom	4-7
	SE1.2	Dog falling out of the window and rescued by boy	
SE2	SE2.1	Boy finding and bit by gopher	8-10 (continuing into SE3)
	SE2.2	Dog interested in and chased by bees	
SE3	SE3.1	Boy searching in tree and knocked off by owl	11-13
	SE3.2	Owl angry and chasing boy away	
SE4	SE4.1	Boy accidently picked up by deer	14-18
	SE4.2	Deer running with boy and boy and dog falling down the cliff	
SE5	SE5.1	Boy and dog sitting in pond	19-21
	SE5.2	Boy and dog hearing something and looking over log	
Resolution	–	Boy and dog finding the missing frog and a whole frog family	22-24

*Story Components:* The presence of each of all the story components was examined, and the total number of story components described was tallied. Scores were calculated separately for the 5 main search episodes (score range of 0-8, one-point scale) and 10 search sub-episodes (score range of 0-13, one-point scale).

*Missing Story Component*: Further, a dichotomous variable was calculated to assess whether all of the story components were included, which allows for a global characterization of whether story structure is complete with all key story plots. Scores were examined separately for the 5 main search episodes and 10 search sub-episodes.

*Setting*: The narrative quality for setting was further evaluated in order to characterize whether the story was grounded in the physical and temporal setting, with 0 = mentioning only the story characters (e.g., ‘there once was a boy and a dog and a frog’); 1 = additionally labeling physical or temporal elements of the scene (e.g., ‘the boy and the dog are looking in a jar at the frog in a bedroom’); and 2 = descriptions of the characters’ interactions with or grounding the characters in the physical or temporal surroundings (e.g. ‘it’s getting late and the boy is climbing into bed’).

*Resolution*: The narrative quality for resolution was also examined, to assess whether the participant was able to infer relationships between the beginning and the end of the story to organize a cohesive story. For the narrative quality of *Resolution*, 0 = no mention of boy and dog finding a frog (e.g., ‘he’s leaving now’); 1 = mentioned that a frog was found but didn’t specify that the frog was the boy’s missing frog in setting (e.g., ‘looks like he found some frogs and now he’s taking one home with him. The end’); and 2 = resolution was in line with story theme, i.e., a frog was found, which was the frog that the boy had lost in setting (e.g., ‘and then they found the frog and the frog had found another frog friend. And they had many frog children. So the little boy decided to leave his frog with the frog family and he took one of the little baby frogs home to raise as a pet’).

*Thematic Coherence*: The thematic coherence was assessed for whether the story theme (i.e., the frog missing and the boy searching) was established and reiterated (‘e.g., Where are you, frog?’, ‘frog is missing and the boy is searching for him’). Whether the story theme was clearly established and mentioned in narrative was assessed, with 0 = no mention of the story searching theme; 1 = mentioned that the frog was missing; 2 = additionally mentioned that the boy was searching for the frog. For whether the story theme was reiterated after the first mention, the presence of descriptions of the story theme after the initial mention was assessed (0 or 1). And the overall *Thematic Coherence* was calculated by summing the two scores (0-3, one-point scale).

#### 2.5.2 Narrative evaluation

Narrative evaluation refers to the narrator’s subjective perspectives and interpretations of relations among story events, and story characters’ thoughts/emotions. Key types of evaluative devices are described below.

*Affect/Cognition*: Affect refers to descriptions of the emotional states (e.g., ‘And to their despair, they found the frog was missing’) and behaviors (e.g., ‘The boy is smiling’) of characters. Cognition refers to descriptions of the cognitive states (e.g., ‘He wondered if the frog was hiding on the other side of the log’) and behaviors (e.g., ‘the dog is chasing the deer to rescue his owner’) of characters that do not have clear emotional valence. The percent of the total amount of affective and cognitive evaluative devices out of total word count was quantified as *Affect/Cognition*.

*Causal Inferences*: The descriptions of causal connections among story events were examined. The causal inferences could be explicit (e.g., ‘He thinks that he will get in trouble because the jar broke’) or implicit (e.g., ‘The boy fell out of a tree when an angry owl shrieked at him’), including causal explanations of story characters’ affective and cognitive states and behaviors (e.g., ‘He’s smiling because he found his frog’), and causal explanations of any behavior or action of the story characters that is not cognitive or affective (e.g., ‘The deer threw them off so they fell into the pond’). The percent of total tallies of all causal inferences out of word count was examined, and the proportions of causal attributions of story characters’ thoughts/emotions, and of other behavioral causal attributions, out of all causal inferences were also separately calculated as Causal Explanations of *Affect/Cognition*, and *Causal Explanations of Behavior*.

Critically, coding of utterances were not mutually exclusive, as a single utterance can reflect both a character’s internal state and explanations of causally related elements within an event. For example, for ‘the dog is chasing the deer to rescue his owner,’ the mental state/cognitive component (e.g., the inferred goal or intention to rescue) was coded under *Affect/Cognition*, while the utterance as a whole was also coded as a causal inference because it provides an explanation for the dog’s actions.

#### 2.5.3 Secondary codes

Additional narrative codes were created post hoc based on coder observations, including *Excessive Detail and Topic Perseveration*.

*Excessive Detail* was coded as a global, subjective rating of overall detail orientation across the entire narrative (0 = adequate, 1 = excessive), reflecting global verbosity or over-elaboration regardless of topic appropriateness or narrative progression, e.g., “So then, at precisely 7:32 in the evening, the little boy, wearing striped pajamas and patterned socks, tiptoed carefully across the creaky wooden floor of his bedroom, holding his stuffed rabbit in his left hand, and peered over the edge of the large, slightly dusty black-and-white glass jar that held the frog, all while making sure not to wake his dog, curled up on the old rug at the foot of his bed”.

*Topic Perseveration* captured repetitive return to a non-central topic (0 = absent, 1 = present) and was coded when a topic occurred three or more times (two repetitions beyond the initial mention). This code excluded the story’s central theme (i.e., the boy searching for the frog) and descriptions of central characters (boy, dog, frog), could occur within or across pages, and reflected repetitive topic-focused narration (e.g., ‘The boy’s floor is very messy. His pajamas are on the floor all crumpled up. They’re sitting there on the floor in his room but it is very very messy. The moon is out and they’re getting ready to go to sleep, but his room is still so messy’).

### 2.6 Coding reliability

Coders were trained on the English and/or Cantonese coding schemes to over 80% reliability, and they were blinded to participant diagnostic status during coding. To assess coding reliability, 35% of English transcripts and 20% of Cantonese transcripts were randomly selected for double coding, with equal representation of diagnosis and sex across groups. For calculation of coding reliability, intraclass correlation coefficients (ICC; 80, 81) were calculated for continuous variables, and percent agreement for nominal variables were calculated. ICCs for narrative evaluation variables (e.g., *Affect/Cognition*) exceeded .77, which indicated good agreement (e.g., 81, 82). Agreement for all nominal narrative variables (e.g., *Thematic Coherence*) ranged 0.83 to 1, except for the *Setting* episode (0.72). The observed agreement is consistent with prior studies using comparable narrative measures (e.g., 59, 78, 83), and may reflect the inherent complexity of higher-order narrative coding.

### 2.7 Gaze processing

**Figure 1:**
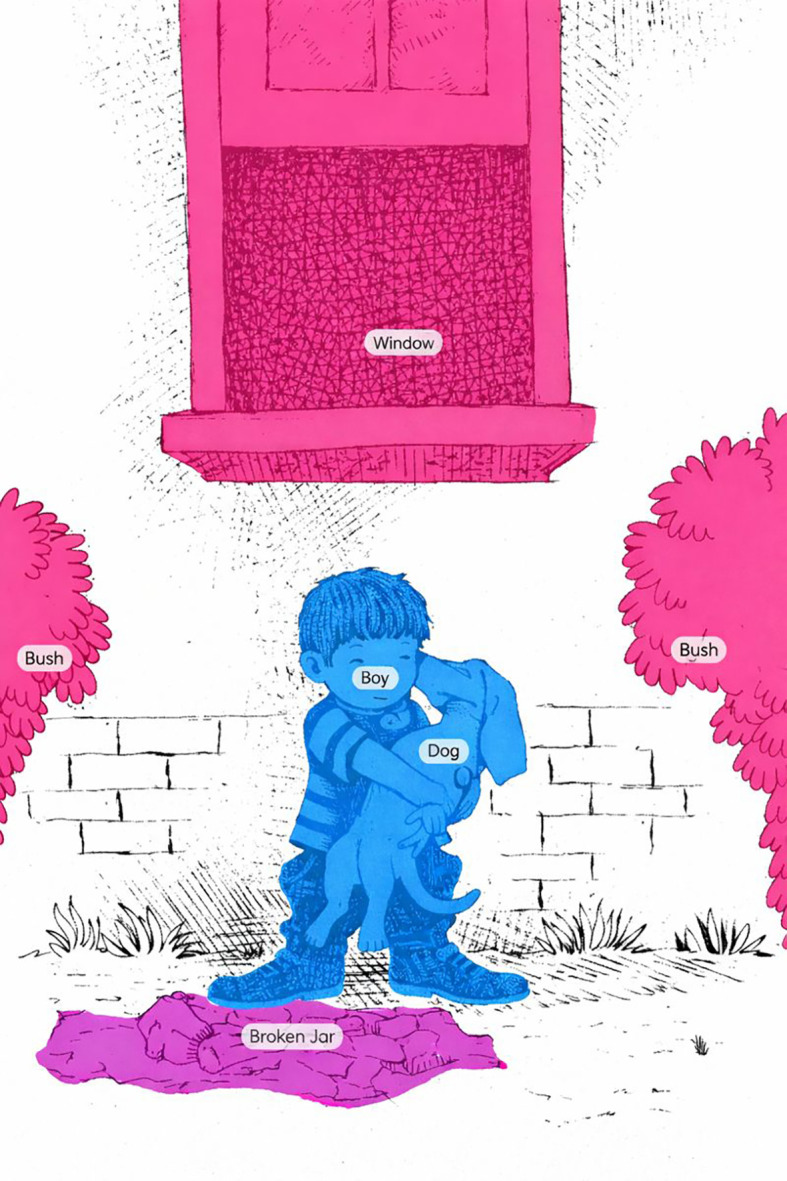
Example Areas of Interest (AOIs) from the Frog Story task. Social AOIs (blue) included the boy and the dog, while non-social AOIs (pink) included the broken jar, window, and bushes.

Quality control criteria for gaze has been reported in prior work in the US sample (33, 57), and on both the HK and the US samples (57), to ensure sufficient gaze tracking during language production. Areas of Interests (AOIs) included social stimuli (i.e., all social story characters and animals), and non-social stimuli (i.e., setting) (see **Figure 1** for example coded AOIs).

Following prior work (33, 57, 58), dynamic gaze patterns summed across all 24 pages were examined, including:

1. Fixation count (i.e., proportions of the number of fixations on each AOI) was used to quantify the distribution of visual attentional focus. The tendency to fixate on social versus non-social stimuli (i.e., *fixation count (%)-social*) was quantified as the proportion of the number of fixations on social stimuli divided by that on non-social stimuli.
2. Refixations: perseverative fixations (i.e., proportions of repeated fixations on the same AOI), and regressive fixations (i.e., proportions of fixations on previously fixated AOI), were measured to understand the visual scanning patterns, and may index local processing and mental disengagement in ASD (e.g., 58, 84). Similarly, the tendency of perseverative and regressive fixations on social stimuli versus non-social stimuli (i.e., *perseverative fixations (%)-social* and *regressive fixations (%)-social*) were calculated by dividing the proportion of the number of perseverative and regressive fixations on social stimuli by those on non-social stimuli, respectively.
3. Transition of fixations: proportions of transitions of fixations between AOIs. This was calculated to measure the patterns of spatial attention allocation (e.g., 58, 85). These variables included transitions of fixations from social to social AOIs (*fixation transition (%)-social to social*), transitions of fixations from non-social to non-social AOIs (*fixation transition (%)-non-social to non-social*), and incongruent transitions of fixations (*fixation transition (%)-incongruent*), which includes transitions of fixations from social to non-social AOIs, and from non-social to social AOIs.

Importantly, prior work has shown that differences in these dynamic gaze patterns, such as perseverative fixations, are observed not only in autistic individuals but also in their parents, suggesting a potential familial or genetic influence (57). Examining these patterns across different cultural contexts allows us to determine which aspects of visual attention are stable and potentially reflect core social cognitive features of ASD, versus which are sensitive to culturally learned norms of attention and communication. If gaze patterns vary across cultures, this may index culturally modifiable aspects of social attention; if patterns remain consistent within families and across cultures, it suggests that the measure may reflect more stable, biologically influenced features of the ASD phenotype. Understanding these distinctions can clarify how dynamic gaze indexes both culturally influenced and core, heritable components of the complex ASD phenotype.

### 2.8 Statistical analyses

#### 2.8.1 Group comparisons on narrative

A series of mixed effects linear regression models were applied for continuous variables (e.g., *Affect/Cognition (%), Story Components Present*) using the nlme package (86) in R (87). All models included culture (HK vs US), diagnosis (ASD vs Control), their interaction term, and covariates (see below) as fixed effects, and participants were included as random effects. Word count was controlled as a covariate for group comparisons for the *Story Components Present* variable to control for the impact of story length on the inclusion of story components (other narrative variables were calculated with word count already controlled as a denominator). Despite known impact of age on narrative abilities (1, 2), age was not included as a covariate due to its match across groups (see **Table 1**) and because age did not significantly correlate with our narrative outcome variables (*rs* ranged from -.09 to .11, *p*s > .13). Verbal IQ data was not available for the HK sample so was not included as a covariate. Chi-square tests were applied for categorical variables (e.g., *Missing Story Component, Thematic Coherence*).

#### 2.8.2 Data reduction for gaze

The principal component analysis (PCA) was applied for z-scored gaze variables using PCA modules implemented in SPSS v24.0. Data from all cultural and diagnostic groups on all fixation variables were included in PCA analyses, including *fixation count (%)-social, perseverative fixations (%)-social*, regressive *fixations (%)-social, fixation transition (%)-social to social, fixation transition (%)-non-social to non-social*, and *fixation transition (%)-incongruent*. The PCA analysis revealed one primary component explaining 76.23% of the variance of the data, and all variables had high loadings (≥ .5) onto the primary component (See **Table 3**). Following previous literature (e.g., 58), the construct was named as ‘social attention’, a greater score on which indicates greater attention towards social stimuli and decreased attention towards non-social stimuli.

**Table 3:** Component matrix from principal component analysis.

Gaze variables	Social attention factor
Fixation Count - Social	.98
Perseverative Fixations - Social	.81
Regressive Fixations - Social	.97
Fixation Transition (%) - Social to Social	.97
Fixation Transition (%) - Non-social to Non-social	-.85
Fixation Transition (%) - Incongruent	-.58

#### 2.8.3 Group comparisons on gaze

A series of mixed effects linear regression models were applied for gaze variables and the PCA social attention component using the nlme package (86) in R (87). All models included culture (HK vs US), diagnosis (ASD vs Control), and their interaction term as fixed effects, and participants were included as random effects. For group comparisons of both narrative and gaze, post hoc pairwise comparisons were performed for significant interaction effects. In addition, pairwise comparisons were conducted even in the absence of significant interaction effects to examine whether diagnostic differences were present across cultures, given our primary research aim of determining whether narrative and gaze differences previously observed in the US group were consistent or distinct in another cultural context, thereby informing culture-specific clinical implications. All post-hoc analyses were conducted using the emmeans package in R, with Tukey adjustments applied to *p* values.

#### 2.8.4 Correlational analysis between narrative and gaze

Exploratory partial Pearson correlational analyses were applied to examine relationships between main narrative variables (i.e., *Affect/Cognition, Story Components Present* (0-13) and *Causal inferences*) and visual attentional patterns (i.e., *PCA-social attention*). Word count was covaried for associations with the *Story Components Present* variable only, as the other narrative variables accounted for word count in their calculations. Correlation analyses were conducted in all groups combined, separately for cultural groups (i.e., HK and US), separately for diagnostic groups (i.e., ASD and Control), and separately for all sub-groups (i.e., HK-ASD, HK-control, US-ASD, and US-control). The Benjamini–Hochberg method (88) was used to correct for multiple comparisons for correlational analyses (*N* = 27), and the false discovery rate was set to .1. For all correlations, the correlation coefficients, *p* values, and Benjamini–Hochberg adjusted *p* values were reported (See **Table 4**).

**Table 4:** Correlations between narrative and social attention component.

Variables	All groups combined	ASD	Control	HK	US	HK-ASD	US-ASD	HK-control	US-control
Social Attention & Affect/Cognition	*r* = .26, *p* = .003**, adjusted *p* = .03*	*r* = .27, *p = .*05, adjusted *p* = .15	*r* = .28, *p = .*01**, adjusted *p* = .05	*r* = .45, *p = .*001***, adjusted *p* = .03*	*r* = .16, *p = .*14, adjusted *p* = .31	*r* = .38, *p = .*16, adjusted *p* = .31	*r* = .22, *p = .*18, adjusted *p* = .32	*r* = .47, *p = .*005**, adjusted *p* = .03*	*r* = .25, *p = .*11, adjusted *p* = .30
Social Attention & Story Components Present	*r* = -.02, *p = .*79, adjusted *p* = .91	*r* = -.03, *p = .*81, adjusted *p* = .91	*r* = .008, *p = .*95, adjusted *p* = .95	*r* = .15, *p = .*29, adjusted *p* = .46	*r* = -.04, *p = .*72, adjusted *p* = .88	*r* = .29, *p = .*32, adjusted *p* = .48	*r* = -.14, *p = .*41, adjusted *p* = .55	*r* = .14, *p = .*43, adjusted *p* = .55	*r* = -.02, *p = .*92, adjusted *p* = .95
Social Attention & Causal inferences	*r* = -.12, *p = .*16, adjusted *p* = .31	*r* = -.03, *p = .*85, adjusted *p* = .92	*r* = -.14, *p = .*23, adjusted *p* = .39	*r* = .31, *p = .*03*, adjusted *p* = .10	*r* = -.31, *p = .*005**, adjusted *p* = .03*	*r* = .62, *p =*.01**, adjusted *p* = .05	*r* = -.24, *p = .*15, adjusted *p* = .31	*r* = .16, *p = .*37, adjusted *p* = .53	*r* = -.33, *p = .*03*, adjusted *p* = .10

*p <.05, **p <.01, ***p <.001***.

## 3 Results

### 3.1 Group comparisons for narrative

**Figure 2:**
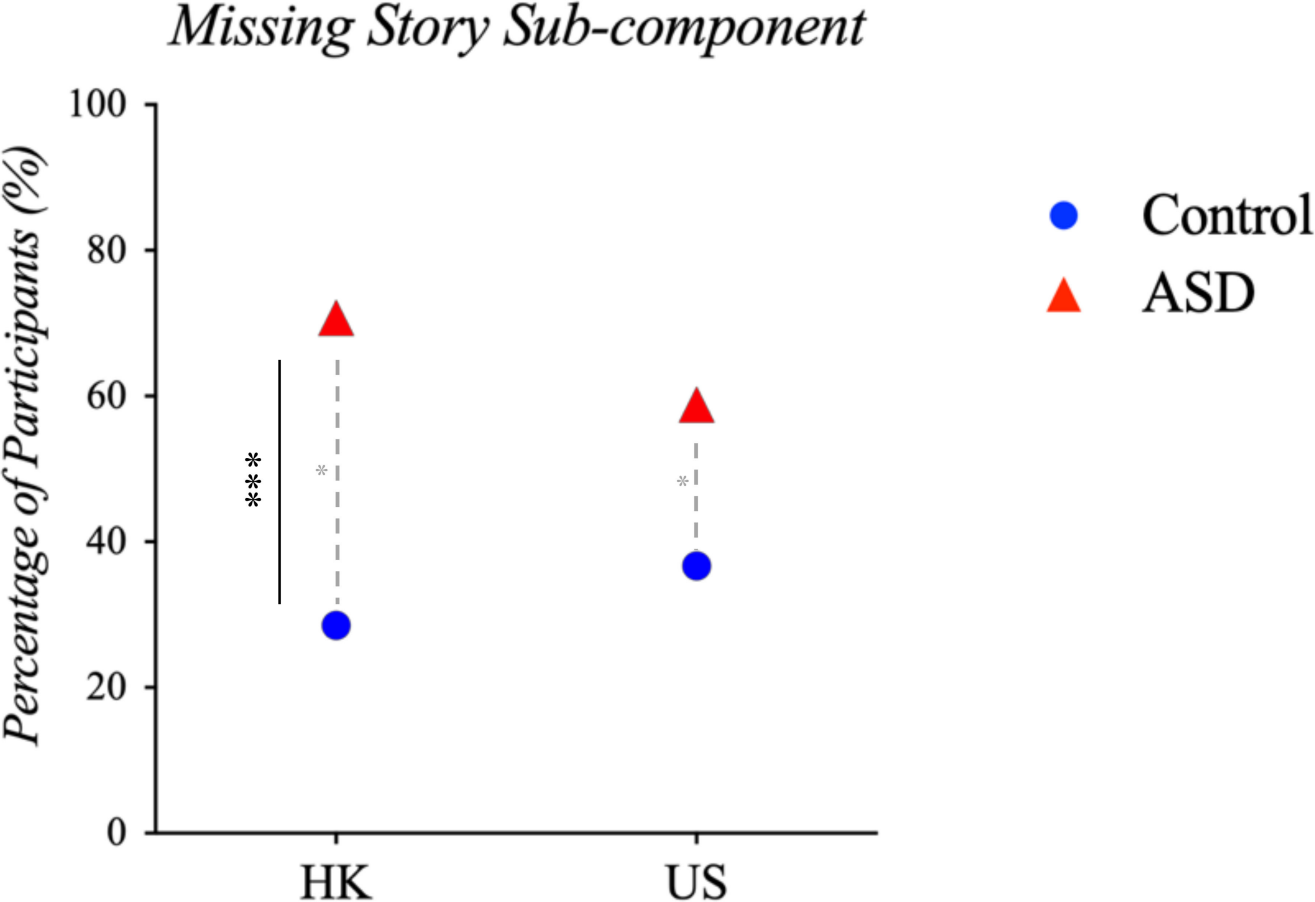
Narrative structure across diagnostic and cultural groups. Across both cultures, a higher percentage of participants in ASD groups (both HK-ASD and US-ASD groups) missed key story sub-components compared to that in control groups (both HK-control and US-control groups). Black bars denote significant overall cultural or diagnostic effect, and dashed grey lines indicate significant pair-wise comparisons. *p <.05, ***p <.001.

A significant main effect of diagnosis was found for narrative structure (*X*^2^(1, *N* = 180) = 10.09, *p* = .001), indicating that autistic participants were more likely to omit key story components than controls. To further examine whether this diagnostic effect was consistent across cultures, we conducted follow-up pairwise comparisons within each cultural group. Diagnostic differences were observed in both the HK-ASD group compared to the HK-control group (*X*^2^(1, *N* = 75) = 5.65, *p* = .02), and the US-ASD group compared to the US-control group (*X*^2^(1, *N* = 104) = 4.3, *p* = .04) (see **Figure 2**).

**Figure 3:**
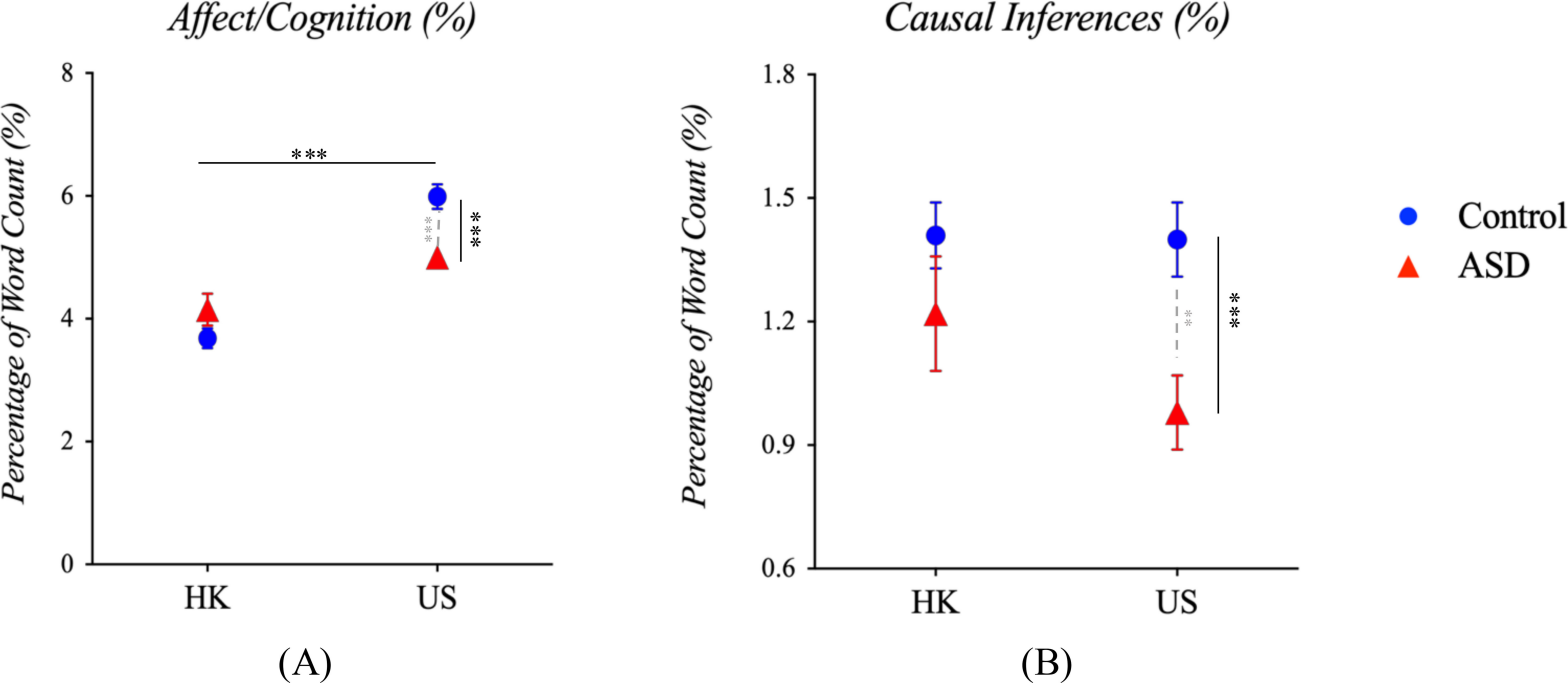
Narrative evaluation across diagnostic and cultural groups. **(A)** Higher percentages of descriptions of cognitive and affective states and behavior were found in US groups than HK groups, and control groups (both HK-control and US-control groups) than ASD groups (both HK-ASD and US-ASD groups), mainly driven by the US-control group higher than the US-ASD group; **(B)** Higher percentages of causal inferences were found in control groups (both HK-control and US-control groups) than ASD groups (both HK-ASD and US-ASD groups), mainly driven by the US-control group higher than the US-ASD group. Error bars represent standard error of the mean (SEM). Black bars denote significant overall cultural or diagnostic effect, and dashed grey lines indicate significant pair-wise comparisons. **p <.01, ***p <.001.

For narrative evaluation skills, independent linear mixed-effects models were conducted for each skill (i.e., affect/cognition and causal inferences). For affect/cognition, significant cultural (*Estimate* = -2.32, *t* = -8.97, *p* <.001), diagnostic (*Estimate* = -.99, *t* = -3.92, *p* <.001), and interaction effects (*Estimate* = 1.46, *t* = 3.58, *p* <.001) were found. Specifically, both HK/US-control groups included a higher percentage of descriptions of thoughts/emotions than ASD groups (both HK-ASD and US-ASD groups). Post hoc pairwise comparisons revealed significant diagnostic difference only between the US-control and the US-ASD groups (*t* = 3.92, *p* = .001), but not between the HK-control and the HK-ASD groups (*t* = -1.47, *p* = .46) (See **Figure 3**).

For causal inferences, both HK-control and US-control groups had greater causal inferences regarding story events (*Estimate* = -.41, *t* = -.3.33, *p* = .001) than ASD groups (both HK-ASD and US-ASD groups). Similarly, post hoc pairwise comparisons revealed the diagnostic difference was only significant between the US-control and the US-ASD groups (*t* = 3.33, *p* = .006), but not between the HK-control and the HK-ASD groups (*t* = 1.18, *p* = .64) (See **Figure 3**). No other significant diagnostic, cultural or interaction effects emerged for narrative skills (*p*s > .06).

### 3.2 Group comparisons for gaze

**Figure 4:**
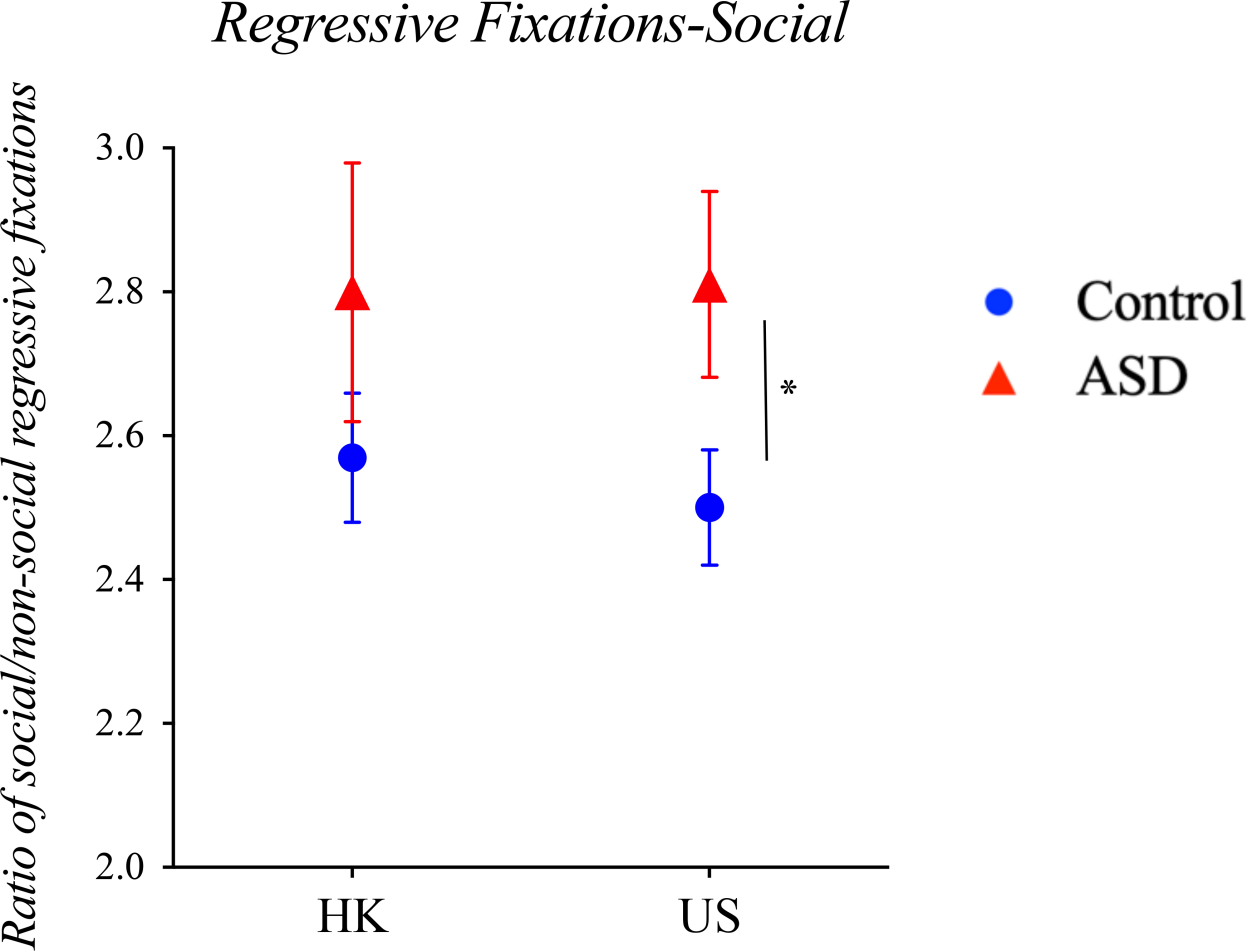
Regressive fixations on social stimuli across diagnostic and cultural groups. The ASD groups (both HK-ASD and US-ASD groups) had a greater tendency of regressively fixating on social versus non-social stimuli than control groups (both HK-control and US-control groups). Error bars represent standard error of the mean (SEM). Black bars denote significant overall cultural or diagnostic effect. *p <.05.

**Figure 5:**
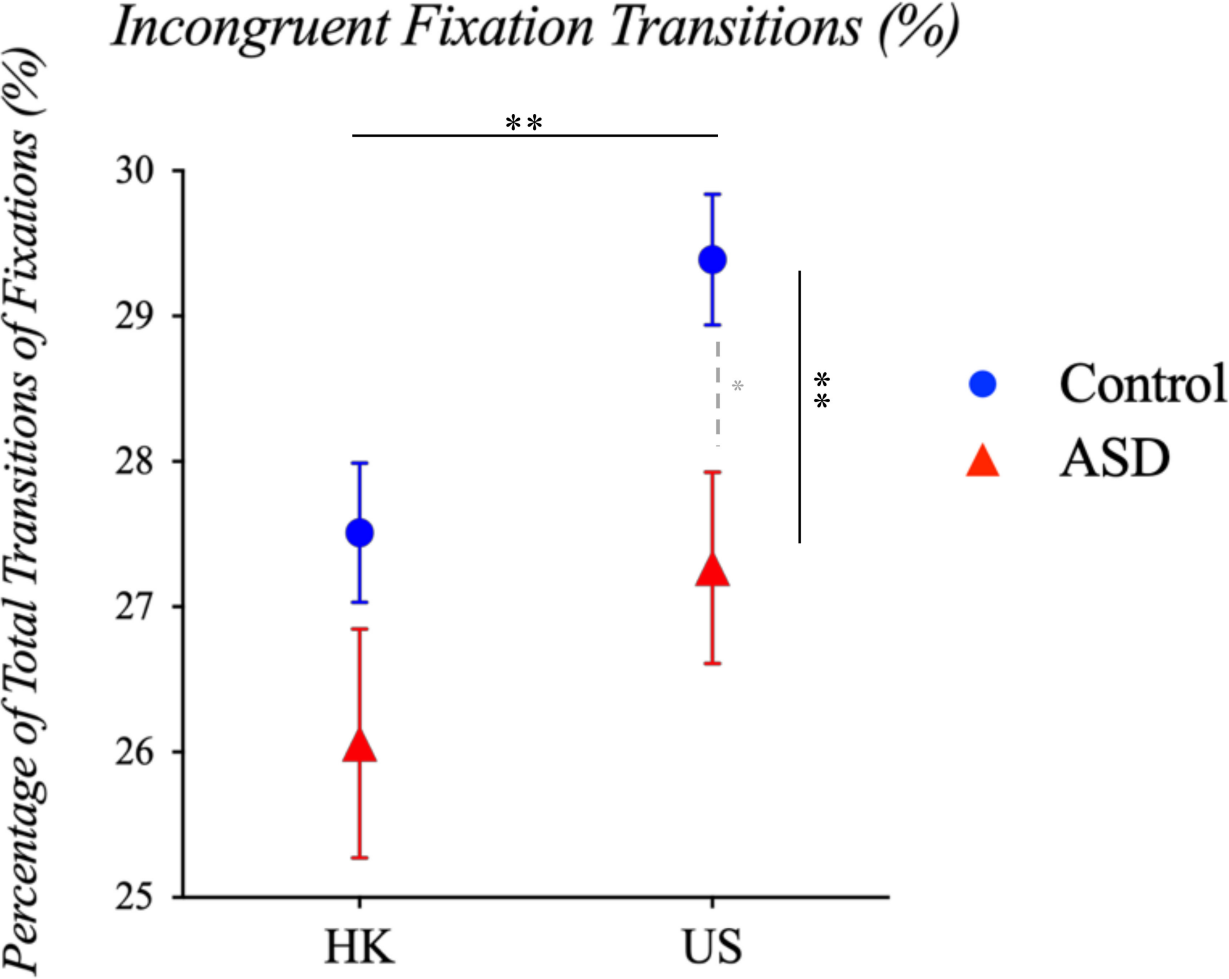
Incongruent fixation transitions across diagnostic and cultural groups. Higher percentages of incongruent fixation transitions were found in US groups than in HK groups, and in control groups (both HK-control and US-control groups) than in ASD groups (both HK-ASD and US-ASD groups), mainly driven by the US-control group higher than the US-ASD group. Error bars represent standard error of the mean (SEM). Black bars denote significant overall cultural or diagnostic effect, and dashed grey lines indicate significant pair-wise comparisons. *p <.05, **p <.01.

Cultural comparisons revealed that both US-ASD and US-control groups had a higher percentage of incongruent fixation transitions (*Estimate* = -1.88, *t* = -2.51, *p* = .01), but a decreased percentage of fixation transitions from non-social to non-social stimuli (*Estimate* = 2.49, *t* = 2.58, *p* = .01), than both HK-ASD and HK-control groups. For diagnostic effects, both HK-ASD and US-ASD groups showed an increased percentage of regressive fixations on social stimuli (*Estimate* = .31, *t* = 2.19, *p* = .03; See **Figure 4**), and a decreased percentage of incongruent fixation transitions (*Estimate* = -2.12, *t* = -2.9, *p* = .004; See **Figure 5**), compared to both HK-control and US-control groups. The diagnostic difference in the percentage of incongruent fixation transitions was primarily driven by the US groups, with the US-control group exhibiting a higher percentage of incongruent fixation transitions than the US-ASD group (*t* = 2.9, *p* = .02). No other significant diagnostic, cultural, or interaction effects emerged for fixation patterns (*p*s > .06). No significant diagnostic, cultural, or interaction effects emerged for the social attention component extracted from PCA (*p*s > .05).

### 3.3 Associations between narrative and gaze

**Figure 6:**
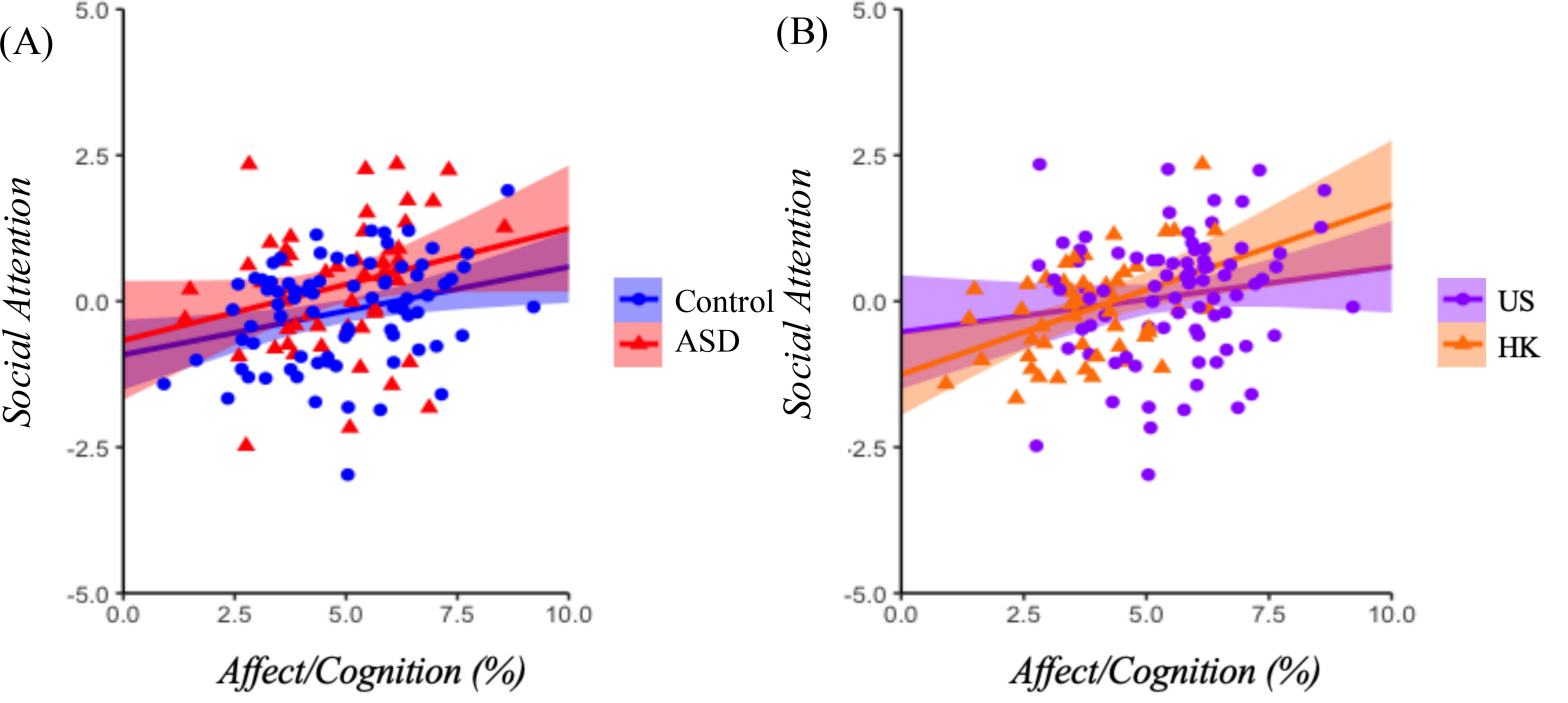
Correlations between narrative and gaze across diagnostic and cultural groups. Greater percentages of descriptions of cognitive and affective states and behavior were correlated with greater scores on the ‘social attention’ component across diagnostic **(A)** and cultural groups **(B)**.

Greater scores on the social attention component extracted from PCA, or greater tendency of gaze towards social stimuli versus non-social stimuli, correlated with greater descriptions of emotions/thoughts of the story characters in all groups combined (*r* = .26, *p* = .003, adjusted *p* = .03), control groups (both HK-control and US-control groups) (*r* = .28, *p* = .01, adjusted *p* = .05), HK groups (*r* = .45, *p* = .001, adjusted *p* = .03), and the HK-control group (*r* = .47, *p* = .005, adjusted *p* = .03) (See **Figure 6**). Cultural differences were detected in the ways in which social attention related to narrative skills. In US groups (*r* = -.31, *p* = .005, adjusted *p* = .03), increased social attention correlated with decreased causal inferences, driven by the US-control group (*r* = -.33, *p* = .03, adjusted *p* = .10). Unlike patterns observed in the US groups, both HK groups showed a pattern where increased social attention correlated with greater causal inferences (*r* = .31, *p* = .03, adjusted *p* = .1), mainly driven by the HK-ASD group (*r* = .62, *p* = .01, adjusted *p* = .05). No other significant correlations emerged (*p*s > .05). See **Table 4** for detailed statistics.

## 4 Discussion

This study investigated the impact of cultural and linguistic background on narrative competence and underlying visual attentional patterns among autistic individuals. Overall, differences in narrative structure and regressive gaze patterns were found in ASD versus controls across cultures, which implicate narrative-related differences in ASD independent of cultural influences and might instead relate to ASD genetics. In contrast, differences in narrative evaluation in ASD were culturally dependent, implicating its malleability to environmental change, and providing insights into the development of culturally sensitive clinical intervention on ASD. These narrative differences were further associated with gaze patterns during narration across cultures, and the variations in visual processing patterns potentially contributed to subsequent differences in narrative content and quality.

### 4.1 Cross-cultural patterns of narrative in ASD

Differences in narrative structure were found in ASD across cultures, where all autistic individuals were more likely to miss key story components compared to respective controls. These findings were consistent with prior literature on differences in narrative structure in ASD in English-speaking western countries (e.g., 6, 16, 17), and the relative independency of narrative structure from influences of cultural backgrounds in non-autistic individuals (e.g., 46). More limited attention to key story elements might be explained by a local processing style common in ASD (e.g., 89, 90). For example, autistic individuals may have a tendency to attend more towards specific features of a scene, rather than establishing a holistic understanding and inclusion of all connected story events (4).

Narrative evaluation skills showed more cultural variability. Specifically, only the US-ASD group had decreased narrative evaluation compared to US-control group. Such differences were not detected in the HK groups. However, both HK groups were found to include significantly fewer descriptions of the protagonists’ thoughts/emotions compared to the US groups, which is consistent with the documented emphasis in Western cultures on cultivating creative thinking and emotional expression that tends to be less prominent in educational practices within Asian cultural contexts (e.g., 42, 43). The overall low level of descriptions of thoughts and emotions observed in both HK groups could therefore explain the lack of diagnostic differences. The culturally variant differences in narrative evaluation have important implications for the environmental (i.e., cultural) basis of narrative differences observed in ASD, indicative of culturally-specific ASD social-communicative difficulties that may be malleable to environmental influences, such as those related to emotion.

These findings underscore the need for a multifaceted, cross-cultural approach to understanding narrative abilities in ASD. The cross-culturally-invariant differences in narrative structure in ASD, together with prior work documenting pragmatic language differences in first-degree relatives of ASD (33, 91–100), may reflect a potential biological influence on narrative differences (95, 100–106). In contrast, the culturally-specific difference found in narrative evaluation in ASD, appears to reflect how cultural values and conventions can shape narrative and the manifestations of narrative differences in ASD. Cross-cultural differences may also provide implications for culturally sensitive clinical practices, e.g., those traits malleable to environmental changes should serve as targets of intervention, and traits targeted might differ across cultures.

### 4.2 Cross-cultural patterns of gaze in ASD

Cross-culturally consistent differences in visual attention were found in ASD, primarily demonstrating patterns of greater rigidity in visual scanning patterns compared to respective controls. Specifically, the ASD group exhibited a marginally higher rate of congruent visual transitions (i.e., fixating from one social stimuli to another social stimuli) coupled with a decreased rate of incongruent transitions (i.e., fixating from a social to non-social stimuli, and the reverse). This, combined with a greater tendency for regressive refixations towards social stimuli, may reflect the well-documented local visual perceptual tendency in ASD (34, 58, 85). Notably, the reduced rate of incongruent transitions was primarily observed among the US-ASD group. A similar trend was observed in the HK-ASD group, althought it did not reach statistical significance, likely due to the small gaze HK-ASD sample size (*n* = 15). Nevertheless, the current findings of culturally-invariant gaze patterns associated with ASD, together with prior work identifying similar gaze differences among clinically unaffected first degree-relatives of ASD (58), may implicate a rigid visual scanning patterns in ASD as a relatively invariant, biologically influenced trait, independent of cultural influence (107, 108).

### 4.3 Associations between gaze and narrative

Differences in social attention were associated with variation in narrative evaluation in ASD across cultures, potentially implicating social attention variability as a source of narrative evaluation differences in ASD. Specifically, decreased social attention in ASD was correlated with decreased descriptions of characters’ thoughts and emotions. Across both cultural groups, when individuals with ASD attended less to the social elements within a story, such as the protagonists’ actions, relationships, emotions, and motivations, they were less likely to talk about these key narrative elements, potentially reflecting differences in conceptual processing of these social components. It’s worth noting that while both HK and US autistic groups exhibited visual attention and narrative associations, only the US group displayed diagnostic group differences in narrative evaluation. This could suggest that US individuals rely more on visual attention for their narrations compared to those in HK, or that the overall lower evaluative narration included by HK groups limited the variation that could be detected between diagnostic groups.

Cultural differences were also found in the relationship between social attention and narrative causal inferences, indicating different social attention patterns may underlie differences in the cognitive processing of causal relationships in narrative. In the US-TD group, decreased social attention was associated with greater causal referencing, whereas in the HK-TD group, increased social attention was associated with greater causal referencing. Similar but not statistically significant cultural differences were also found in ASD groups (both HK-ASD and US-ASD groups). These disparate relationships between gaze and narrative might be linked to variations in visual preferences in different cultures. For example, individuals from Eastern collectivist cultures may pay more attention to setting information, whereas those from Western individualist cultures may prioritize focal salient stimuli (e.g., 49–51). Therefore, individuals from both cultures may need to focus on information they typically overlook to gather insights into the connections between story events and predict their causal relations (e.g., this may involve increased attention on salient social story characters for HK groups and on non-social elements for US groups). The findings identified culturally specific strategies for utilizing visual information to shape narratives, highlighting how cultural factors intersect with gaze differences as fundamental elements underlying narrative competence. Moreover, these results suggest the importance of culturally tailored clinical interventions that target gaze as a mechanistic factor for enhancing narrative and broader social communicative skills in ASD.

The mechanisms and neural underpinnings associated with narrative differences and gaze patterns supporting narrative in ASD across cultures have not been directly examined in prior literature. Emerging evidence suggests that differences in altered responses across visual, auditory, and somatosensory pathways and multisensory processing may contribute to the social communicative differences observed in autistic individuals (109–113). At the neural level, regions central to social perception such as the superior temporal sulcus (STS) and its functional interactions with other social-brain nodes, show differences in responsivity and connectivity in ASD (e.g., 114–117), which could underlie differences in how social information is attended to and interpreted. Importantly, recent eye-tracking and behavioral work links these altered gaze dynamics (e.g., increased refixations) with downstream language and narrative outcomes (34), suggesting a cascade in which early sensory-perceptual and attentional biases impede the encoding of characters’ actions, mental states, and causal relations that support coherent storytelling. Cultural contexts may influence the expression of these biological mechanisms. Cross-cultural differences in social norms, attention to social cues, and storytelling conventions can shape patterns of gaze and narrative in both non-autistic and autistic individuals (57). In our study, differences observed between US and HK participants may reflect the interaction between underlying neurophysiological processes and culturally shaped attentional priorities, emphasizing the need for culturally sensitive interpretations of gaze and narrative behaviors in ASD. Future research should examine how cultural norms interact with sensory and neural pathways to influence social communication and narrative development in ASD.

### 4.4 Limitations and future directions

First, although we applied language-related inclusion criteria by excluding US participants with verbal IQ below 80 and HK participants with impaired language development as assessed by the HKCOLAS, and controlled for word count in analyses, the study did not include identical, cross-linguistically matched measures of cognitive and language abilities. Given the methodological challenges of comparing or matching language ability across cultural groups, future studies should consider developing new approaches for matching participants on language abilities or including comparable language measures as covariates to more fully rule out language-related confounds. Additionally, while many individuals in the US society are monolingual, the majority of people from HK are multilingual. Given that multilingualism can impact elements of cognition and language (e.g., 118), future work should include an objective measure of bilingualism and multilingualism to control for this potentially confounding variable. Future studies should also aim to more closely match groups on autism severity to enhance comparability across cultural contexts and ensure that observed differences are not driven by variability in clinical presentation. Additionally, although the frog story narrative task’s wordless format facilitates cross-linguistic comparison, it may not fully capture culturally specific storytelling conventions. The story reflects narrative themes and settings common in Western children’s literature (e.g., individual child protagonists, forest exploration, and pet ownership as central plot elements), which may align more closely with narrative schemas familiar to Western participants. Culturally diverse narrative stimuli, e.g., stories depicting everyday family interactions or characters and settings representing multiple cultural contexts, may provide a more culturally generalizable assessment of narrative abilities for the HK sample. Future studies using culturally grounded narrative materials are needed to test the generalizability of these findings across contexts. Also, the storytelling task is highly structured, and it will be important for future studies to also study different communicative contexts with different cognitive and social demands, and in more naturalistic contexts, such as conversation. Another limitation of the current study is that information about co-occurring conditions (e.g., ADHD, anxiety, or other neurodevelopmental or psychiatric disorders) was not available for all participants across the US and HK samples. Future studies should include detailed assessments of co-occurring conditions to better characterize the clinical profiles of autistic participants and understand how these may interact with communication and attentional patterns reported in this study. Lastly, while the small HK-ASD sample may limit statistical power, this study provides a first step in characterizing potential language and cultural differences in cross-linguistic samples, using a quasi-experimental design to examine how language and culture may modulate autistic phenotypes within a verbally fluent subset. The sample targeted autistic individuals with lower support needs (e.g., verbal IQ ≥ 80 for US groups; adequate language development for HK groups), who nonetheless show clinically significant social communication differences, allowing investigation of cross-cultural variation in narrative and gaze patterns. Despite modest sample size, controlled comparisons across two geographical and linguistic contexts yielded medium effect sizes for key contrasts: diagnostic differences in narrative structure between HK-ASD and HK-controls (*φ* = .27) and culture × diagnosis interactions for affect/cognition (*r* = .26), supporting meaningful group differences. Future research should include larger and more diverse samples to improve generalizability, particularly given the heterogeneity in cognitive and language abilities among individuals with ASD. Including participants with a broader range of language fluency and larger sample sizes will help determine whether the observed patterns generalize across the wider autistic population and across different cultural and linguistic contexts.

## 5 Conclusion

In summary, the current study revealed both cultural similarities and differences in narrative and associated gaze patterns among ASD groups from the US and HK. The results may shed light on how cultural and linguistic backgrounds, and underlying biological factors shape narrative differences in ASD, implicating future studies further understanding the etiology of social-communicative difficulties in ASD. Findings may be useful clinically, in identifying traits potentially more malleable for focus of intervention, as well as for targeting culturally specific social communication profiles in ASD.

## Data Availability

Data from both sites can be made available upon reasonable request (HK data: Prof. Patrick Wong (p.wong@cuhk.edu.hk; US data: Prof. Molly Losh, m-losh@northwestern.edu).

## Footnotes

1 Given differences in language preferences among stakeholder communities, both person-first and identity-first terminology were used when referring to participants with autism (ASD; including HK-ASD and US-ASD groups). In some languages (e.g., Chinese), terminology equivalent to “disorder” is required for grammatical and conventional usage (e.g., 自閉症 / 孤獨症), and does not carry the same connotation as in English.
